# Comprehensive Treatment Module for Medically Unexplained Physical Symptoms – A Guide for General Practitioners

**DOI:** 10.7759/cureus.16263

**Published:** 2021-07-08

**Authors:** Aastha Goel, Piyush Ranjan, Kamal B Klanidhi, Koushik Sinha Deb, Siddharth Sarkar, Tanveer Kaur, Anamika Sahu, Upendra Baitha, Avinash Chakrawarty, Arvind Kumar

**Affiliations:** 1 Internal Medicine, All India Institute of Medical Sciences, New Delhi, IND; 2 Medicine, All India Institute of Medical Sciences, New Delhi, IND; 3 Internal Medicine: Geriatrics, All India Institute of Medical Sciences, New Delhi, IND; 4 Psychiatry, All India Institute of Medical Sciences, New Delhi, IND; 5 Psychology, All India Institute of Medical Sciences, New Delhi, IND; 6 Internal Medicine • Geriatric Medicine, All India Institute of Medical Sciences, New Delhi, IND

**Keywords:** mups, medically unexplained physical symptoms, management, approach, mus, somatization disorder, somatoform disorders

## Abstract

Purpose: This study was aimed to develop a comprehensive treatment module that the general physician can use to manage patients with Medically Unexplained Physical Symptoms (MUPS) at a primary care level.

Methods: This comprehensive module was developed after a literature review followed by its validation by a two-step Delphi technique with experts from internal medicine, psychiatry, and clinical psychology.

Results: The developed module for the patients with MUPS includes case diagnosis, initial evaluation, strategy for referral, and a comprehensive treatment module. The comprehensive treatment module includes symptom discussion, rapport establishment, physical health counselling, stress management, cognitive strategies for symptom control, comorbidities treatment, and medical management.

Conclusion: The developed module has unique features, such as intensive sessions with these patients, a patient-specific treatment strategy, and a holistic approach incorporating pharmacological and non-pharmacological interventions. General Practitioners across the world can use this comprehensive treatment module for the management of patients with MUPS.

## Introduction

Medically unexplained physical symptoms (MUPS) are the symptoms that have little or no underlying organic basis of pathology. These symptoms are either inconsistent or out of proportion to the underlying disorder [1]. Studies have shown that the healthcare facilities, mostly medical OPDs, are often burdened with MUPS patients as these patients preferentially use medical services over mental health services [2,3]. A study conducted in a tertiary care hospital in North India found that approximately 24.6% of patients presenting to OPD had MUPS [4]. Patients with MUPS have decreased quality of life, and most of the domains of the World Health Organisation Disability Score (WHO-DAS) are affected in these patients [5]. Besides, the caregivers of these patients perceive similar levels of the subjective and objective burden as caregivers of patients with chronic depression and schizophrenia [6]. 

Patients with MUPS are often dissatisfied with the treatment offered by the doctors and are also hesitant to seek help from a psychiatrist or a mental health professional [7,8]. Therefore, it becomes essential for the general physician to learn optimal management strategies for adequate and appropriate management of MUPS. This study has been planned to develop and validate a module that would be useful for general Practitioners in treating this common and essential condition.

## Materials and methods

The Comprehensive Treatment Module (CTM) was developed systematically using a scientifically approved methodology. A systematic literature search was carried out to formulate the CTM for MUPS. The two-step Delphi method was conducted between October and December 2020 to generate a consensus among experts. 

Panel selection

Ten experts participated in the process of development of the module. The experts had considerable experience in managing patients with MUPS and were interested in developing a treatment module. The panel consisted of professionals from the field of internal medicine, psychiatry, and clinical psychology. The characteristics of the panel experts are summarized in the table below (Table 1).

**Table 1:** Characteristics of panel experts

Parameters	(n=10)
Age (years)	42.1 +/- 5.62
Sex	
Male	8 (80%)
Females	2 (20%)
Current role	
Professor/Additional professor	3 (30%)
Associate professor	4 (40%)
Assistant professor	2 (20%)
Psychologist	1 (10%)
Educational qualification	
MD/MS	7 (70%)
PhD	3 (30%)
Speciality	
Medicine	4 (40%)
Psychology	3 (30%)
Psychiatry	3 (30%)
Years working in the field	
More than 10 years	9 (90%)
6-9 years	1 (10%)

Review of literature

An in-depth literature review was done to look for the pre-existing information in the field of treatment modalities for MUPS. The search results were used to define the construct of the module. PubMed, Cochrane, and Wiley databases were used for literature search with the following search string: ('Medically Unexplained Symptoms' OR 'Medically Unexplained Physical Symptoms' OR 'MUPS' OR 'MUS' OR 'unexplained physical symptoms') AND ( Psychotherapy OR 'Cognitive Behaviour Therapy' OR 'Behaviour* Therapy' OR 'Psychological Intervention' OR mindfulness OR 'Relaxation Therapy'). Studies published in English from 2000 to 2020 were used for this paper. Two authors AG and TK independently reviewed the papers, and discrepancies were resolved through third-party adjudication (PR and SS).

Data extraction and development of statements

The relevant information was collected from the selected studies regarding non-pharmacological strategies to manage MUPS. The information was extracted and was used to develop consensus statements. A total of 32 statements were compiled (Table 2), which were circulated amongst all expert panel members for round 1 of voting.

**Table 2:** List of statements circulated to all members of the expert panel for voting MUPS- Medically unexplained physical symptoms

Module 1: Ascertainment of diagnosis and problem assessment
Detailed history should be taken in patients with MUPS to rule out organic pathology
Thorough physical examination should be performed in patients with MUPS to rule out organic pathology
Appropriate investigations should be conducted in patients with MUPS to rule out organic pathology as deemed necessary
Extensive investigations should be avoided unless the physician has a compelling reason for the same
Referral to speciality services should be sought for those patients where the treating physician has a doubt in the diagnosis of MUPS
Module 2: Psychoeducation and physical counselling
The physician should work on rapport establishment with the patient.
Patients with MUPS will benefit from a discussion of their symptoms.
Patients with MUPS will benefit from clarification of misconceptions about the symptoms and disease.
Patients with MUPS will benefit from clarification of misunderstandings about medical care.
Dietary counselling should be done for patients with MUPS.
Patients should be encouraged to participate in graded physical activity.
MUPS patients should be encouraged to participate in social activities.
Patients should be encouraged to keep records of daily activities.
Module 3: Stress management
For each patient, an attempt should be made to identify the precipitating factor
Patients should be encouraged to participate in activities they used to find pleasurable
Patients should be encouraged to regularize their sleep schedule (sleep hygiene)
Breathing exercises for relaxation will be beneficial in patients with MUPS
Autogenic training will be beneficial for patients with MUPS.
Module 4: Cognitive strategies of symptom control
Cognitive-behavioral therapy is an important tool for the management of MUPS
Problem solving treatment is a practical approach that can be delivered by general practitioner.
Teaching problem solving techniques to MUPS patients will be beneficial

Round 1

The list of statements was circulated amongst the panel members via email and a clear explanation of the study's objectives and instructions for the members' participation. Each expert was asked to vote by marking "agree," "disagree," or "neutral" beside each statement. Experts were also given a choice to provide their comments and suggest additional items. The response for each statement was entered into an excel sheet, and the consensus level was set at 70% (i.e., an agreement between 7 out of 10 experts). In other words, if 7 out of 10 experts agreed on a statement, it would be accepted for the next round. The statements which did not meet the consensus level of 70% were further modified as per the feedback provided and were presented to the experts in round 2.

Round 2

The same voting method, as described for round 1, was used for round 2. However, this was accompanied by the collection/knowledge of group scores and additional comments. This was done so that the experts could reflect upon the group results and change their opinion accordingly. The final responses were analyzed and used to construct a CTM.

## Results

Development of statements

For the development of the treatment module, information was compiled from studies narrowed in the systematic review of the literature. This was used to generate 32 statements which are summarised in Table 2.

Round 1

The response rate for round 1 was 100%. Thirty-one out of the total 32 statements reached a consensus level of more than 70%. Out of these 32, 14 statements were agreed upon by all the experts. The views reaching 100% consensus are described in the table below (Table 3).

**Table 3:** Statements reaching 100% consensus in Round 1 MUPS- Medically unexplained physical symptoms

A detailed history should be taken in patients with MUPS to rule out organic etiology
Thorough physical examination should be performed in patients with MUPS
The physician should work on rapport establishment with the patient
Patients should be encouraged to participate in graded physical activity
MUPS patients should be encouraged to participate in social activities
Patients should be encouraged to keep a record of daily activities
Patients should be encouraged to participate in activities they used to find pleasurable
Cognitive-behavioral therapy is an important tool for the management of MUPS
Teaching problem-solving technique to MUPS patients will be beneficial
Coping mechanisms will be beneficial in patients with MUPS (E.g.- emphasizing working around the symptoms rather than working on the symptoms)
Patients should be made aware of a realistic sense of symptom control
Appropriate medical management of comorbidities should be done
The review of progress in previous sessions should be performed at each subsequent session
Patients should be taught how to maintain learned skills and coping strategies

The statement which did not get 70% consensus was, "Problem-solving treatment is a practical approach that a general practitioner can deliver." The following modifications were suggested: "With adequate exposure and expertise to the practitioner," "With some training and active supervision," "Only if the practitioner is interested and willing to spend time." The statement was then modified into "Problem-solving treatment is a practical approach that can be delivered by a general practitioner provided there is adequate training and motivation" and circulated for round two of Delphi.

Round 2

The response rate for round 2 was 100%. All statements were circulated with accompanying comments, previous responses of the expert, and the mean of all the scores. All 32 statements reached more than 70% consensus in round 2. Sixteen out of the 32 statements received 100% consensus in round 2 (this included four new statements that achieved 100% consensus after round 2, whereas the consensus decreased to 90%). The four new statements which reached 100% consensus are tabulated below (Table 4).

**Table 4:** Statements reaching 100% consensus in Round 2 MUPS- Medically unexplained physical symptoms

Appropriate investigations should be conducted in patients with MUPS to rule out organic pathology (as deemed necessary by the treating physician)
Referral to specialty services should be sought for those patients where the treating physician has a reasonable doubt in the diagnosis of MUPS
Patients with MUPS will benefit from the clarification of misconceptions about the symptoms and disease
An individual treatment plan should be drawn for possible relapse

After completing the steps mentioned above, a module for management of patients with MUPS by physicians was developed, which is described below:

Case definition

MUPS are any principal somatic complaints reported by the patient for which no definite medical diagnosis could be found even after many physical examinations and appropriate investigations. Even if a disease is under consideration, the reported severity of the symptom(s) is disproportionate for the diagnosed disease [9].

Initial evaluation

Baseline Investigations

This includes investigations like i) Complete blood counts, Erythrocyte Sedimentation Rate(ESR), Serum C-Reactive Protein(CRP), ii) Fasting blood sugar, iii) Liver function tests/Kidney function tests iv) Thyroid Function Test, v) Lipid Profile, HbA1C vi) Chest X-Ray (PA view) vii) Electrocardiography (ECG) viii) Urine routine microscopy ix) Stool routine microscopy.

Clinical Examination

A thorough clinical examination of all the patients with MUPS is mandatory. The general physical examination includes examining pulse rate, measuring blood pressure, respiratory rate, temperature, and examining pallor, icterus, cyanosis, clubbing, lymphadenopathy, and pedal edema. A system-wise examination should be conducted with specific emphasis on the system of involvement, i.e., if a patient is presented with complaints of predominant bowel symptoms, the complete abdominal examination should be conducted along with the examination of other systems examination as deemed appropriate.

Specialized Investigations

These tests are recommended depending upon the symptoms of the patient and clinical suspicion of the treating physician: i) Antinuclear antibody (suspicion of autoimmune disorder) ii) Ultrasound whole abdomen (bowel predominant symptoms) iii) Creatine phosphokinase (persistent myalgias) iv) Serum Vitamin B12 levels/folate levels (neurological symptoms) v) Viral markers (high degree of clinical suspicion) vi) Non-contrast CT head (new-onset headache/suspicion of organic etiology).

The decision of Imaging investigations should be based on the symptoms of the patient and the clinical suspicion. For example, suppose the patient is presented with persistent headaches with symptoms suggestive of an organic etiology or red flag signs initially. In that case, a non-contrast CT scan/MRI of the head can be advised. Other modalities like PET scans can be advised as and when indicated. It is emphasized that if clinicians doubt the diagnosis, one should investigate further or seek speciality referral before labelling the patient as a case of MUPS.

Diagnosis

MUPS is essentially a diagnosis of exclusion. The diagnosis can be established when the patient has persistent somatic complaints without a reasonable explanation of clinical examination or investigations. The Mini-International Neuropsychiatric Interview (MINI) can be used to screen for any associated psychiatric comorbidity.

When to seek a referral

The bizarre and multiple symptoms pose difficulty in its appropriate evaluation. An appropriate referral from specialists should be sought if the red flag symptoms are found during the evaluation. For instance, if a patient complains of persistent headache with abdominal discomfort with chest discomfort, they can be considered medically unexplained if no organic basis is found on relevant examinations and investigations. However, suppose the patient has a headache with persistent vomiting and blurring of vision. In that case, there is a high likelihood of organic pathology (raised intracranial pressure), and such patients should be referred to a neurologist for further evaluation.

Another significant group of patients may have complaints about the gastrointestinal system in the form of persistent abdominal discomfort and dyspepsia. Such patients should also be interviewed for red-flag symptoms like altered bowel habits, blood in stools, weight loss, or documented fever. If any doubt remains in the mind of the treating physician, a speciality referral can be sought before labelling the symptoms as medically unexplained. This, however, needs to be reiterated to the patients that even after seeking speciality care, they should report to the general practitioner for regular health visits.

Comprehensive Treatment Module

The components of the module will be divided amongst various sessions. i) Symptom discussion, ii) Rapport establishment, iii) Physical health counselling, iv) Stress management, v) Cognitive strategies for symptom control, vi) Management of comorbidities, vii) Medical management

Symptom Discussion

It is important to spend time with the patient to elaborate on his/her symptoms. Encourage him/her to reflect on what it may feel like to live with these symptoms and how it impacts his/her life. Very often, these patients, after being labelled as "MUPS," feel ignored and unheard. Therefore, ample time should be given to them to detail their symptoms [10,11]. The patients can be encouraged to maintain a symptom diary, which can be reviewed at every session to see for any improvement or worsening [12,13].

Rapport Establishment

Open-door Policy: It should be clear to the patient that the doctor will be available for him/her even after the sessions are over. An emergency contact number may be provided to the patient to contact during increased stress [14].

Non-abandonment: The patients should be ensured that they will not be abandoned at any point in time.

Goal Setting: It is recommended to set realistic goals after discussion with the patient. The patient may be given a worksheet, where completion of each goal will be emphasized as being integral to symptom control. These goals include practising relaxation techniques daily, daily physical activities, and practising hobbies if any. Family members who accompany the patient should be suggested to ensure that the patients engage in the things mentioned above [15].

Physical Health Counselling

Dietary counselling: A diet plan can be given to each patient after consultation with the dietician to promote healthy eating behaviour. The assurance of compliance to the dietary plan may be done through family members visiting the physician with the patient. Further, the patient should be counselled at each step that adherence to healthy habits is integral to improvement in his/her general condition.

Routine physical activities: Discuss with the patient the activities he/she participates in for physical, emotional, spiritual, intellectual, and social health and well-being. The patient should identify the activities they currently participate in. Ask the patient if they are satisfied with their current participation in health-promoting activities. Several scales are available for use for assessment of participation in health-promoting activities, which include Activities of Daily Living Scale, Instrumental Activities of Daily Living Scale, Health Promoting Activities Scale (HPAS), Global Physical Activity Questionnaire, Saltin- Grimby Physical Activity level Scale [16,17]. Scales are available to check for adherence to diet and physical activity-related advice [18,19].

Stress Management

Patients should be taught various relaxation techniques, which can be taught to the patients to be practised at least once a day and during increased distress [20,21],

Deep breathing consists of taking several deep breaths, holding them for five seconds, and then exhaling slowly;

Mindfulness meditation is a self-directed practice for relaxing the body and calming the mind; 

Autogenic training, which consists of imagining a peaceful environment and comforting bodily sensations.

Cognitive Strategies for Symptom Control

Defocusing: Explain to the patient the importance of symptom reattribution, i.e., diverting the thought process from the symptoms to other activities of their interest. Re-focusing from negative thoughts (inflexible and unnecessary) to positive thoughts should be taught. E.g., if the patient says, "I am never going to get better. I am always going to be in pain." Such negative thoughts need to be re-focused into more positive ones like "I can do activities that will help me feel better. My symptoms are not permanent. I do not have a serious medical condition."

Acceptance: This strategy may be useful to reduce the severity of symptoms. Practising a bargaining approach may be useful. The doctor may counsel that even if the likelihood of getting a complete remission of the symptoms is remote, the treatment will reduce it by 30%.

Cognitive distortion: Specific assumptions about an illness, symptoms, and health behaviours (such as frequent medical consultations) often arise from past experiences, including medical treatment of family and friends. These assumptions provide anxiety and vulnerability to misinterpretation of changes in appearance or bodily sensations [22]. Therefore, various cognitive techniques can manage the patients' negative thoughts related to the illness and promote positive health behaviour.

An alternative explanation of symptoms: This technique can be used to control the severity of the symptoms. Both doctor and the patient can be asked to hold their arm at a right angle to their body for two minutes. Depending on how strong they are, they will start to notice pain in the arm muscles. This example can demonstrate that physical sensations and pain can result from muscles' use unusually.

Normalizing symptoms: Frequently, patients report an increased frequency of symptoms due to selective attention that is also present in the general population. For example - a patient may complain of difficulties with memory and concentration (i.e., forgetting what he wanted to buy when he got to the shop) with a normal neurological examination. Questions regarding similar symptoms can be asked from accompanying persons, and it can be demonstrated that such symptoms are reported by a large number of the general population.

Management of Comorbidities

MUPS is not just the absence of an organic disease. At times, the organic disease is present, and the unexplained symptoms are disproportionate to the existing disease. i.e., if a patient with well-controlled hypertension complains of persistent headache not otherwise explained, it can be attributed to MUPS. In such cases, it becomes important to recognize the underlying disease, like hypertension. Both lifestyle and pharmacological management should be initiated to manage this case.

Medical Management

i) Selective serotonin inhibitors (SSRI), ii) Selective noradrenaline and serotonin inhibitors (SNRI), iii) Tricyclic antidepressants (TCA). These are prescribed in a low dose initially and slowly titrated upward. Both tricyclic antidepressants and SSRIs are almost equally effective in the treatment of MUPS. However, SSRIs may be preferred over tricyclic antidepressants due to the favourable side effect profile. Escitalopram is very effective in predominant MUPS and multi-somatoform disorders and can be used as a first-line drug [23]. The brief outline of the sessions and the time duration has been summarized in the table below (Table 5).

**Table 5:** Brief outline of Comprehensive Treatment Module For MUPS MUPS- Medically unexplained physical symptoms

Area	Session and duration	Content	Structure
Module 1: Problem Assessment	Session 1	Initial assessment	Interview and investigation-based approach, Detailed history, Investigations, Assessment of psychological construct by scales
Module 2: Psychoeducation and physical counselling	Session 2 (25 min)	Rapport establishment, Symptom discussion, Psycho-education, Self-monitoring (diary/journaling), Dietary Counselling, Routine physical activities, Reassurance, and Validation of symptoms (normalizing symptoms)	Psychoeducation, Handout, Daily dairy sheets, List and detail of physical activities, Homework sheets
Module 3: Stress Management	Session 3 : (25 min): Face to face session Session 4 : (15 min) [Telephonic]	Review of symptom diary, Review of Health Promoting Activities, Pleasant activities, Breathing exercise, Systematic relaxation training, Sleep hygiene	Ideas for pleasant activities, Handouts, and homework sheets
Module 4: Cognitive strategies for symptom control	Session 5-6 : (20 min)	Review of a symptom diary, Review of Health Promoting Activities, Defocusing, Identifying Cognitive distortion, Cognitive restructuring	Handouts and homework sheets
Module 5: Management of comorbidities (Optional) Relapse prevention and review of progress	Session 7: (15 min)	Review of symptom diary (5 min), Health Promoting Activities (5 min), Other medical and psychiatric comorbidities management (5 min)	List of disease and medicine, Road map to follow a plan
		Tips for maintaining improvement, Review of goals achieved.	Handouts

## Discussion

MUPS patients are often considered "difficult" by General practitioners. These patients usually present with many bizarre symptoms, which forces the physician to advise multiple investigations and seek referral services early in outpatient visits [24].

This module is the first of its kind that can be used in primary care/OPD setup. A comprehensive approach for managing patients with MUPS is expected to be more beneficial than the conventional method. This module was built, modified, and validated by standard methods. After an extensive review of the literature, it was developed that most of these patients often have their first consultation with a primary care physician. There is no other standard management strategy for patients with MUPS that is both patient-centric and time-intensive, which general care practitioners can use. Studies have found that self-help plays a vital role in treating these patients [25]. The module attempts to define a clear set of investigations - both general and specialized investigations- that should be ordered in patients with MUPS. This module also helps to understand when the patient should be referred to as speciality services.

This module consists of seven sessions that are to be completed over four months. The module incorporates carefully decided components that will be reinforced during each session. The first session will allow a thorough understanding of the patients' symptoms, establishment of diagnosis by relevant examination and investigations. The second session will include establishing rapport with the patient, which is often overlooked in general practice. The purpose of rapport establishment is to trust the treating physician so that the patient is comfortable discussing the problem [26]. It includes a review of the patient's condition and whether or not they are engaged in pleasure and general health activities. Numerous studies have described exercise and physical health benefits on mental well-being, which we will improve [27]. The third and fourth sessions include stress management strategies [28]. These strategies may be performed with the patient during the session. The fifth and sixth sessions include cognitive strategies of symptom control, discussed in detail in the module [29]. The last session includes an overall assessment of the progress made by the patient. The patients should be encouraged to keep a diary to monitor their progress. Further, compliance with the basic stress management techniques offered will be ensured through the patient's attendant. The management of coexisting comorbidities should be taken care of, along with appropriate pharmacological therapy.

## Conclusions

The comprehensive treatment module has been developed after reviewing available literature and evidence bearing in mind that most of these patients often visit a primary care physician. This module will be useful in treating patients with MUPS in resource-limited settings. This can be used in the developed world after making necessary subtle changes. This module was vetted by a group of experts consisting of internists, psychiatrists, and clinical psychologists. A randomized controlled trial should be conducted to evaluate its efficacy.
